# Extracellular matrix stiffness controls osteogenic differentiation of mesenchymal stem cells mediated by integrin α5

**DOI:** 10.1186/s13287-018-0798-0

**Published:** 2018-03-01

**Authors:** Meiyu Sun, Guangfan Chi, Juanjuan Xu, Ye Tan, Jiayi Xu, Shuang Lv, Ziran Xu, Yuhan Xia, Lisha Li, Yulin Li

**Affiliations:** The Key Laboratory of Pathobiology, Ministry of Education, Norman Bethune College of Medicine, Jilin University, Changchun, 130021 People’s Republic of China

**Keywords:** Matrix stiffness, Mesenchymal stem cells, Differentiation, Integrin α5

## Abstract

**Background:**

Human mesenchymal stem cell (hMSC) differentiation into osteoblasts has important clinical significance in treating bone injury, and the stiffness of the extracellular matrix (ECM) has been shown to be an important regulatory factor for hMSC differentiation. The aim of this study was to further delineate how matrix stiffness affects intracellular signaling through integrin α5/β1, FAK, and Wnt signaling, subsequently regulating the osteogenic phenotype of hMSCs.

**Methods:**

hMSCs were cultured on tunable polyacrylamide hydrogels coated with fibronectin with stiffness corresponding to a Young’s modulus of 13–16 kPa and 62–68 kPa. After hMSCs were cultured on gels for 1 week, gene expression of *alpha-1* *type* *I* *collagen*, *BGLAP*, and *RUNX2* were evaluated by real-time PCR. After hMSCs were cultured on gels for 24 h, signaling molecules relating to integrin α5 (FAK, ERK, p-ERK, Akt, p-Akt, GSK-3β, p-GSK-3β, and β-catenin) were evaluated by western blot analysis.

**Results:**

Osteogenic differentiation was increased on 62–68 kPa ECM, as evidenced by *alpha-1 type I collagen*, *BGLAP*, and *RUNX2* gene expression, calcium deposition, and ALP staining. In the process of differentiation, gene and protein expression of integrin α5/β1 increased, together with protein expression of the downstream signaling molecules FAK, p-ERK, p-Akt, GSK-3β, p-GSK-3β, and β-catenin, indicating that these molecules can affect the osteogenic differentiation of hMSCs. An antibody blocking integrin α5 suppressed the stiffness-induced expression of all osteoblast markers examined. In particular, *alpha-1 type I collagen*, *RUNX2*, and *BGLAP* were significantly downregulated, indicating that integrin α5 regulates hMSC osteogenic differentiation. Downstream expression of FAK, ERK, p-ERK, and β-catenin protein was unchanged, whereas Akt, p-Akt, GSK-3β, and p-GSK-3β were upregulated. Moreover, expression of Akt and p-Akt was upregulated with anti-integrin α5 antibody, but no difference was observed for FAK, ERK, and p-ERK between the with or without anti-integrin α5 antibody groups. At the same time, expression of GSK-3β and p-GSK-3β was upregulated and β-catenin levels showed no difference between the groups with or without anti-integrin α5 antibody. Since Akt, p-Akt, GSK-3β, and p-GSK-3β displayed the same changes between the groups with or without anti-integrin α5 antibody, we then detected the links among them. Expression of p-Akt and p-GSK-3β was reduced effectively in the presence of the Akt inhibitor Triciribine. However, Akt, GSK-3β, and β-catenin were unchanged. These results suggested that expression of p-GSK-3β was regulated by p-Akt on 62–68 kPa ECM.

**Conclusions:**

Taken together, our results provide evidence that matrix stiffness (62–68 kPa) affects the osteogenic outcome of hMSCs through mechanotransduction events that are mediated by integrin α5.

**Electronic supplementary material:**

The online version of this article (10.1186/s13287-018-0798-0) contains supplementary material, which is available to authorized users.

## Background

Human mesenchymal stem cells (hMSCs) mediate the repair and regeneration of various adult tissues via self-renewal and multilineage differentiation potential [1]. In vitro, hMSCs require biological cues for their proliferation and differentiation, which are largely controlled by cell–cell interactions, insoluble factors (such as extracellular matrix (ECM)), soluble cytokines, and growth factors [2]. Extensive research has demonstrated that the osteogenic differentiation of hMSCs can be artificially regulated by modifying their microenvironment, such as through increased ambient dexamethasone levels or UV irradiation [3, 4]. However, these treatments are far less effective for bone injuries in vivo; therefore, a clear understanding of the mechanical forces that induce hMSC differentiation into osteoblast is needed. Cells are subjected to a majority of these mechanical forces when interacting with the ECM or other adjacent cells [5, 6]. In particular, the effect of ECM on hMSCs could be attributed to the substrate’s stiffness [5], which is one of the several factors that contribute to wide variations in the rigidity of human tissues and organs [7]. Thus, the stiffness provided by ECM as a biological scaffold plays a key role in cellular fate [8].

Ample evidence suggests that ECM stiffness as a physical factor in the microenvironment can regulate MSC differentiation into nerve cells, chondrocytes, myocytes, and osteoblasts in two-dimensional culture conditions. For instance, polyacrylamide hydrogels with variable stiffness attributed to *alpha-1 type I collagen* (*COL1A1*) content showed effective induction of osteogenic hMSC differentiation [8], characterized by *RUNX2* upregulation; however, the mechanism by which this occurs remains unclear.

ECM stiffness regulates cell differentiation primarily via integrin interactions. Integrins are a family of heterodimeric surface molecules that regulate intracellular and extracellular signaling pathways to affect the survival [9], migration [10, 11], and differentiation [12, 13] of hMSCs. For example, the integrin α5/β1 heterodimer plays an important role in the molecular induction of osteogenic hMSC differentiation. Individually, integrin α5 can increase *RUNX2* and *COL1A1* expression while increasing mineralization [12], whereas integrin β1 is believed to be the primary mediator of osteogenic differentiation in response to mechanical stimulation [14]. Moreover, integrin α5 is upregulated during chemical-induced osteogenic differentiation of hMSCs and plays a critical role in this process by regulating focal adhesion kinase (FAK)/extracellular-related kinase (ERK) and mitogen-associated protein kinase (MAPK) signaling [12, 15–19]. Activation of PI3K would be important in mediating osteogenic differentiation [20], as it is involved in the regulation of MSC proliferation and osteogenic differentiation [21]. The mechanism of differentiation and selection of stem cells is still not well understood.

Downstream integrin signaling plays an important role in osteogenesis. In particular, FAK displays a specific regulatory role in stem cell behavior [10] in that it can induce hMSC osteogenic differentiation and promote bone calcium absorption [22–24]. Insulin-like growth factor 1 (IGF-1) and other growth factors can activate PI3K/Akt, resulting in downstream mTORC1/S6 K1-mediated signaling and apoptotic resistance. Moreover, osteogenic differentiation of hMSCs is characterized by increases in mitochondria number and morphological changes [25], which are often associated with the PI3K/Akt pathway. The Wnt signaling pathway is involved in a variety of cell activities [26, 27]. Integrins could regulate the differentiation of cells [28, 29] through the Wnt signaling pathway. Wnt5a enhances integrin mRNA and protein expression, and further regulates MSC osteogenic differentiation [30]. Similarly, some reports have shown that integrin α7 and integrin α2 can induce skeletal stem cells and dentin-like cells differentiated into osteoblasts respectively [31]. Thus, Wnt signaling and integrins engage in crosstalk during differentiation.

Integrin α5/β1-mediated signaling thus plays a key role in chemical-induced MSC differentiation into osteoblasts, albeit through an unclear mechanism. Therefore, the present study investigated the impact of ECM stiffness on integrin α5/β1 and its downstream signaling molecules; and osteogenic differentiation of hMSCs with the goal of developing novel therapeutic approaches to promote bone formation.

## Methods

### Cell culture and characterization

HMSCs were maintained in Dulbecco’s modified Eagle medium supplemented with 10% FBS, 10 ng/ml bFGF, 100 U/ml penicillin, and 100 μg/ml streptomycin. The growth medium was changed every 3 days. Only passage 3–6 hMSCs were used for experimental studies. After using inhibitor of Akt (10 mM TCBN, MCE) to treat cells on 62–68 kPa ECM for 24 h, they were detected by western blot analysis. All experimental procedures were approved by the ethics committee of Jilin University and conformed to the regulatory standards.

Detection of surface markers of hMSCs was determined using flow cytometry and immunofluorescence staining. hMSCs were collected and washed with prewarmed (37 °C) PBS three times and then fixed with 4% paraformaldehyde for 15–20 min. After washing three times, the cells were then blocked with 1% BSA in PBS for 30–40 min and incubated with 10 μg/ml anti-CD34, anti-CD44, anti-CD45, anti-CD90, or anti-CD105 (Millipore, USA) for 1 h. The probed samples were subsequently washed three times with PBS and then examined by flow cytometry (BD FACSCalibur) or fluorescence microscope (Olympus CKX41SF).

The osteogenic differentiation of hMSCs was induced in osteogenic medium containing 0.1 μmol/L dexamethasone, 50 μg/ml ascorbic acid, 10 nM vitamin D3, and 10 mmol/l β-glycerophosphate. The differentiation of hMSCs into adipocytes was induced in adipogenic medium containing 1 μM dexamethasone, 100 μg/ml (0.45 mM) IBMX, 10 μg/ml insulin, and 0.1 mM indomethacin. The differentiation-inducing medium was changed every 2 days. BMMSCs were used at passage 3 for all experiments.

### Growth of cells

Cell growth curves were recorded using a counting method. Cells at passage 6 in the logarithmic growth phase were plated on a 24-well plate at a density of 10,000 cells per well. The number of cells per well was counted each day, and the growth curve was produced.

### Oil Red O and Alizarin Red S staining

For detection of lipid droplets, hMSCs cultured in adipogenic medium for 2 weeks were fixed with 4% paraformaldehyde for 10 min and then stained with Oil Red O for 10 min at room temperature. For characterization of the mineralized matrix, hMSCs cultured in osteogenic medium for 3 weeks were fixed with 3.7% paraformaldehyde and stained with 1% Alizarin Red S solution in water for 10–15 min at room temperature. The cells were observed under an inverted phase-contrast microscope (Olympus CKX41SF).

### Cell karyotype analysis

For karyotype analysis, cells were treated with colchicine and digested with trypsin. Thereafter, the mixture was centrifuged and the pellet was collected, which was washed once with PBS. This was followed by treatment with low hypotonic KCl (0.075 M). The cells were then incubated; the incubation time differs with cell type, ranging from 20 to 40 min. The cells were centrifuged at 1500 rpm for 5 min. The supernatant was discarded, leaving behind a small volume (1/10) to mix the pellet. The fixative solution was added slowly dropwise until the tube was full to resuspend the pellet. Again, the cells were centrifuged at 1500 rpm for 5 min and the supernatant was discarded; the step of adding fixative solution and centrifuging was repeated. The pellet was resuspended in a few drops of the fixative and 1–2 drops of it were placed on a slide kept at 0 °C in a water bath. The slide was removed from the water bath and dried in air or by rapid overheating. Giemsa staining was performed, followed by microscopic observation.

### ECM fabrication

Tunable ECM was prepared based on a previous report [8]. Briefly, 8% acrylamide (Sigma-Aldrich, St. Louis, MO, USA) and varying concentrations of bis-acrylamide (0.1%, 0.3%, 0.5%, and 0.7%) (Sigma-Aldrich) were mixed and then polymerized with tetramethylethylenediamine (TEMED) and ammonium persulfate (AP) (Sigma-Aldrich) on aminosilanized 12-mm or 24-mm-diameter coverslips. We used previous methods to make ECM gels [32]. The polyacrylamide coverslips were subsequently coated with 0.2 mg/ml *N*-sulfosuccinyimidyl-6-(4′-azido-2′-nitrophenylamino) hexanoate (sulfo-SANPAH; ThermoScientific, Waltham, MA, USA) dissolved in 10 mM HEPES (pH 8.5) and exposed to 365-nm ultraviolet light for 70 min. Subsequently, the coverslips were incubated in fibronectin solution (1 μg/cm^2^; Sigma-Aldrich, USA) overnight at 4 °C prior to cell plating. The elastic modulus for each concentration of polyacrylamide hydrogel was measured with a biomechanical testing machine under contact load at a strain rate of 0.5 mm/s.

### CCK8 cytotoxicity assays

To assess cell viability, 2000 cells were plated in 100 μl in each well of a 96-well plate and incubated for 24 h in a humidified incubator (at 37 °C, 5% CO_2_). The seeded cells were cultured in the presence of hydrogel extract solution for 24 h, 48 h, or 72 h before the addition of CCK8 solution (10 μl/well). Plates were incubated for 1 h and then the absorbance at 450 nm was measured with a microplate reader.

### Scanning electron microscopy

Cells were fixed in 2.5% glutaraldehyde for 2 h, rinsed three times in PBS buffer for 15 min each, incubated in osmium acid for 2 h at 4 °C, rinsed again, rinsed three times in PBS for 10 min each, and then dehydrated with a standard ethanol gradient. The samples were incubated in tert-butyl alcohol overnight at −20 °C, freeze-dried with a vacuum, and sputter-coated with gold powder. Samples were then examined under a scanning electron microscope (Hitachi S-3400 N).

### Alkaline phosphatase staining

Cells were fixed in cold propanol, rinsed with water four times, and incubated in a solution consisting of 3% β-glycerophosphate, 2% barbiturate, 2% CaCl_2_, and 2% MgSO_4_ in distilled water for 4 h at 37 °C. The samples were then rinsed three times, incubated with 2% cobalt nitrate for 5 min, rinsed four times, and treated with 1% ammonium sulfide. The processed slides were rinsed, dried, and sealed prior to imaging.

### Confocal microscopy and flow cytometry

To assess integrin α5 and integrin β1 distribution, hMSCs were washed with PBS three times, fixed with 4% polyformaldehyde for 20 min, blocked with 1% BSA in PBS for 30 min, and then incubated with 5 μg/ml integrin α5 or β1 antibodies (AB1928 for integrin α5, MAB2252 for integrin β1; Millipore, Billerica, MA, USA) for 1 h. Nuclei were stained with 4′,6-diamidino-2- phenylindole (DAPI, US Everbright Inc.) and then examined by confocal microscopy (Olympus FV 1200) or flow cytometry (BD FACSCalibur).

### FITC-Phalloidin staining of F-actin

After hMSCs were cultured on the ECM for 24 h, they were washed three times in prewarmed PBS for 10 min. Then 4% paraformaldehyde was fixed for 10 min at room temperature, washing three times. Phalloidin working solution (5 μg/ml) stained the cells for 60 min at room temperature. The probed samples were subsequently washed three times with PBS and then examined by confocal microscopy (Olympus FV 1200).

### Gene expression analysis

Total RNA was extracted with TRI reagent (Takara, Tokyo, Japan) and used to synthesize first-strand cDNA using a Primescript RT reagent kit (Takara). The quantitative real-time reverse transcription polymerase chain reaction (qRT-PCR) was then used to determine the relative expression of the osteogenic markers *COL1A1*, *RUNX2*, and * BGLAP*. The PCR thermal profile consisted of an initial 10 min at 95 °C, followed by 40 cycles of 95 °C for 15 s and 60 °C for 1 min. Gene expression was normalized to that of *GAPDH* and fold change was calculated with the comparative Ct method. Primers were obtained from Sangon Biotech (Shanghai, China). Primer sequences are presented in Table 1.

**Table 1:** Primers used for the quantification of markers

Gene	Forward (5′–3′)	Reverse (5′–3′)
*ITAGA5*	GACAGGGAAGAGCGGGCACTATGG	GTCCCTTCCCGGCCGGTAAAACTC
*ITGB1*	TGCCAGCCAAGTGACATAGAGA	ATCCGTTCCAAGACTTTTCACAT
*COL1A1*	GCCAAGACGAAGACATCCCA	GGCAGTTCTTGGTCTCGTCA
*RUNX2*	TTACCCCTCCTACCTGAGCC	TGCCTGGGGTCTGAAAAAGG
*BGLAP*	ATGAGAGCCCTCACACTCCT	CTTGGACACAAAGGCTGCAC
*GAPDH*	CTTTGTCAAGCTCATTTCCTGG	TCTTCCTCTTGTGCTCTTGC

### Integrin blocking experiments

hMSCs were incubated with antibody against integrin α5 blocking antibody (ab78614; Abcam, Cambridge, UK) in serum-free media for 30 min and seeded onto gels in serum-free media at 5 × 10^3^ cells per cm^2^. Cells were allowed to attach for 12 h, and then the gels were washed with PBS to remove loosely adherent cells. The cells were cultured for 24 h for analysis by western blotting, and for 1 week for qRT-PCR.

### Western blot analysis

Cells were lysed in buffer containing protease and phosphatase inhibitors (Dingguo, Beijing, China). Proteins were quantified, separated by electrophoresis on 8% polyacrylamide gels, and then transferred to polyvinylidene fluoride (PVDF) membranes. Membranes were blocked and probed with antibodies for integrin α5 (Millipore), integrin β1 (Millipore), ERK1/2 (Cell Signaling, Danvers, MA, USA), phospho-ERK1/2 (Cell Signaling), FAK (04–591; Millipore), Akt (Cell Signaling), p-Akt (Cell Signaling), GSK-3β (Cell Signaling), p-GSK-3β (Cell Signaling), β-catenin (Cell Signaling), and GAPDH (Sigma-Aldrich) overnight at 4 °C, followed by secondary antibodies on the next day. Immunoreactive bands were visualized by chemiluminescence.

### Statistical analysis

Data represent the mean ± standard error of at least three samples. Statistical significance was determined by two-way ANOVA analysis with Tukey’s post-hoc testing. *P* < 0.05 and *P* < 0.01 were considered statistically significant.

## Results

### The characteristics of hMSCs

**Fig. 1:**
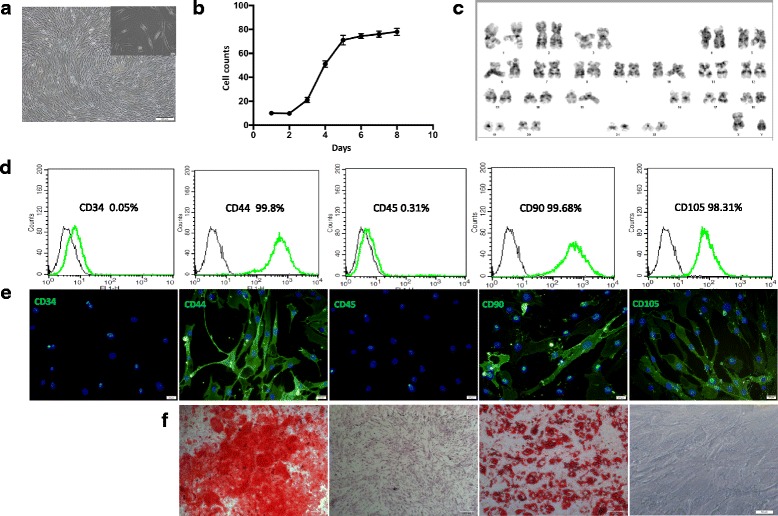
Identification of hMSCs. **a** Morphological appearance of third-passage hMSCs. Scale bar = 200 μm (inset 20 μm). **b** Logarithmic proliferation of cells. **c** Chromosome karyotype analysis of cells. **d** hMSC surface markers evaluated through flow cytometric analysis. **e** Immunofluorescence performed using monoclonal antibodies. **f** Differentiation of hMSCs into adipocytes and osteoblasts. Scale bar = 100 μm. *n* = 3

After 1 week, cells isolated from bone marrow adhered to culture dishes. The cells principally formed bipolar spindle-like cells after they grew to passage 3. When confluence reached 90%, the cells exhibited a spiral shape (Fig. 1a and Additional file 1: Figure S1A). The latency of subcultured cells was approximately 72 h, and the logarithmic proliferation period was 3–7 days (Fig. 1b and Additional file 1: Figure S1B). In the karyotype analysis, the chromosomes of 20 mitotic cells were counted, and most of the cells had 46 chromosomes. Chromosome karyotype analysis was performed on five of the mitotic cells, and no abnormality was found. The statistical results were 46, XY, for normal healthy male human cells that can be used for experimental research. These cells were used in our subsequent experiments (Fig. 1c and Additional file 1: Figure S1C). hMSCs at passage 3 were strongly positive for hMSC markers, such as CD44, CD90, and CD105, and negative for CD34 and CD45, results shown by flow cytometry and immunofluorescence staining analyses (Fig. 1d, e and Additional file 1: Figure S1D, E). Furthermore, the isolated cells showed the potential to differentiate into adipogenic and osteogenic lineages after culturing in induced medium. Cells contained a lot of Oil-Red-O-positive lipid globules after 2 weeks induced with adipogenic medium. Similarly, calcium deposits stained by Alizarin Red were detected in osteogenic hMSCs after 3 weeks induced with osteogenic medium. In sum, our results demonstrated that the hMSC we used in our experiments were multipotent and responsive to differential stimuli (Fig. 1f and Additional file 1: Figure S1F).

### ECM preparation and analysis

**Fig. 2:**
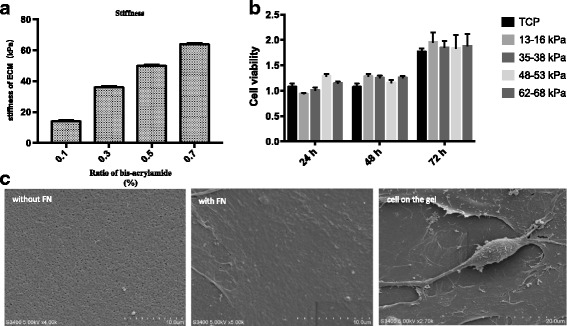
Extracellular matrix (ECM) hydrogel substrates for cell growth. **a** Effect of bis-acrylamide ratio on ECM stiffness measured with a power reactor. **b** Cells cultured with gel solution extract and cell viability assessed 24 h, 48 h, or 72 h later. **c** Scanning electron microscopy images of gels without or with 0.2 mg/cm^2^ fibronectin (FN) coating (scale bar = 10 μm) and FN-coated substrate with cells (scale bar = 20 μm). *n* = 3. ECM extracellular matrix, TCP tissue culture dishes

ECM samples were prepared by mixing 8% acrylamide with various concentrations of diacrylamide to prepare materials with variable stiffness (13–16 kPa, 35–38 kPa, 48–53 kPa, and 62–68 kPa) (Fig. 2a). Because acrylamide monomers can affect cell proliferation, we extracted solution from the incubated gels and treated hMSCs for 24 h, 48 h, and 72 h; however, no significant differences were found when compared to normal controls (Fig. 2b). Scanning electron microscopy (SEM) of the prepared ECM revealed a smooth gel surface with nanoscale pores. The gel was subsequently coated with 0.2 mg/cm^2^ fibronectin to overcome it, as cell adhesion molecules can bind to the RGD fragment of fibronectin [33]. SEM analysis of cells plated on the fibronectin-coated substrate showed clear pseudopod extensions that facilitate substrate binding (Fig. 2c). Thus, these data demonstrate that cells can survive on the formulated gel matrix with no apparent toxicity.

### ECM stiffness induced the osteogenic differentiation of hMSCs

**Fig. 3:**
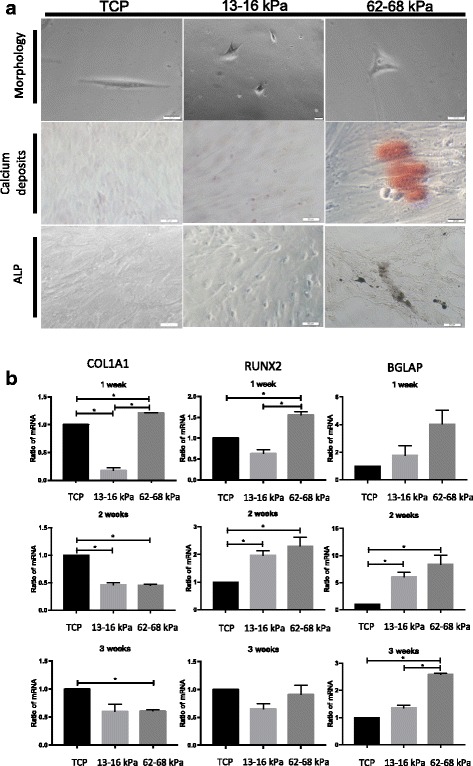
Osteogenic differentiation of hMSCs cultured on 62–68 kPa ECM. **a** Cells plated on indicated substrates for 1 week and then subjected to morphological analysis, Alizarin Red and alkaline phosphatase (ALP) staining. Scale bar = 20 μm. **b** Expression of osteoblast markers *COL1AI*, *RUNX2*, and *BGLAP* measured by qRT-PCR after 1, 2, and 3 weeks of culture. *P < 0.05. *n* = 3. TCP tissue culture plates, COLIAI alpha-1 chains of type I collagen, RUNX2 Runt related transcription factor 2

Morphological analysis of hMSCs plated on ECMs with differing stiffness demonstrated an increased potential for osteogenic differentiation on 62–68 kPa ECM after culturing for 24 h; the cells took on a polygonal morphology typical of osteoblasts. In comparison, weaker 13–16 kPa ECM appeared to promote adipogenesis, as cells gradually retracted from their usual long spindle shape (Fig. 3a and Additional file 1: Figure S2). F-actin was smaller, shorter, and irregularly arranged when cells cultured on 13–16 kPa ECM, whereas F-actin stretched and arranged regularly on 62–68 kPa ECM (Additional file 1: Figure S3). Moreover, intracellular Alizarin Red-positive calcium nodules and alkaline phosphatase (ALP)-stained crystals were readily observed after 1 week on 62–68 kPa ECM, indicating matrix mineralization (Fig. 3a). hMSCs were cultured with osteogenic medium on the 13–16 kPa ECM, 62–68 kPa ECM, and TCP for 1 week, and all three groups had calcium deposition (Additional file 1: Figure S4), suggesting stiffness induction could be changed by biochemical cues. Subsequent mRNA expression analysis revealed a marked increase in the expression of the osteoblast markers *COL1A1* at 1 week and *RUNX2*at 1-2 weeks on 62–68 kPa ECM, respectively (Fig. 3b). In addition, the osteogenic marker *BGLAP (osteocalcin)* was upregulated at weeks 2–3 of culture. Collectively, these results support that culture on 62–68 kPa ECM induced hMSC differentiation into osteoblasts.

### Altered distribution of integrin α5/β1 during stiffness induced osteogenic differentiation of hMSCs

**Fig. 4:**
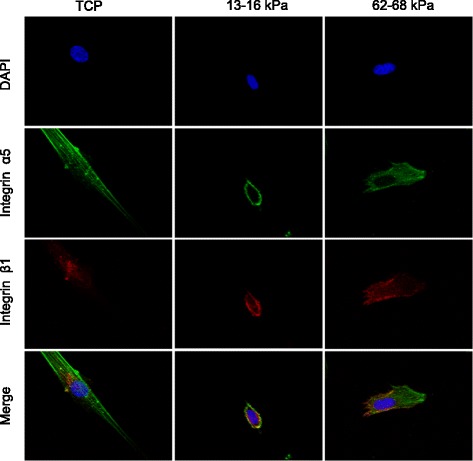
Identification of α5/β1. Distribution of integrin α5/β1 during hMSC culturing on 13–16 kPa ECM, 62–68 kPa ECM, and TCP detected by confocal microscopy using monoclonal antibodies. *n* = 3. TCP tissue culture plates, DAPI 4′,6-diamidino-2-phenylindole

Integrin α5/β1 acts as a starting molecule for adhesion of plaque, as it is located on the cell surface and is involved in cell adhesion, migration, and differentiation. Confocal immunofluorescence microscopy showed that integrin α5/β1 was primarily localized to the cell membrane under standard culture conditions, but acquired a more cytoplasmic distribution after culture on a stiff substrate. Integrin α5 was located on the surface of the cell membrane, while integrin β1 was distributed both on the cell surface and in the cytoplasm in the 13–16 kPa ECM, 62–68 kPa ECM, and TCP groups (Fig. 4). Different strains have different cell fates, and the distribution of integrin α5/β1 differed in cells with different matrix stiffness; thus, the distribution of integrin is involved in cell fate regulation. Integrin α5/β1 binds around the nucleus, facilitating the signaling of downstream signaling molecules.

### Increased expression of integrin α5 on 62–68 kPa ECM

**Fig. 5:**
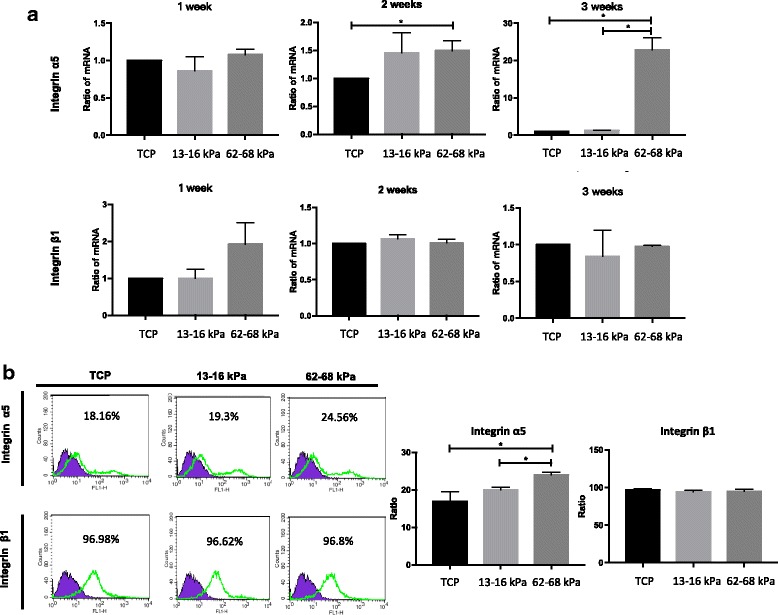
*Integrin* *α5*/*β1* expression analysis of mRNA and surface protein during hMSC culturing on 13–16 kPa ECM, 62–68 kPa ECM, and TCP. **a** mRNA expression analysis of *integrin α5* and *integrin* *β1* at weeks 1–3. **b** Integrin α5/β1 surface protein expression determined by flow cytometry. *P < 0.05. *n* = 3. TCP tissue culture dishes

Quantitative real-time PCR (qRT-PCR) analysis indicated that *integrin* *α5* expression increased after culturing on 62–68 kPa ECM for 2–3 weeks, whereas *integrin β1* had an upregulated trend only during week 1 (Fig. 5a). Consistently, surface integrin α5 protein expression was significantly higher in hMSCs cultured on 62–68 kPa ECM as compared to the other two groups after 24 h, whereas no marked differences were observed for integrin β1 (Fig. 5b). Together, integrin α5 plays a role during the process of stiffness-induced osteogenic differentiation and integrin β1 has no significant effect in the process. We then examined expression of active integrin β1 and found that it was more highly expressed on 13–16 kPa ECM (Additional file 1: Figure S5).

### Increased protein expression of molecules downstream of integrin α5/β1 and Wnt/β-catenin on 62–68 kPa ECM

**Fig. 6:**
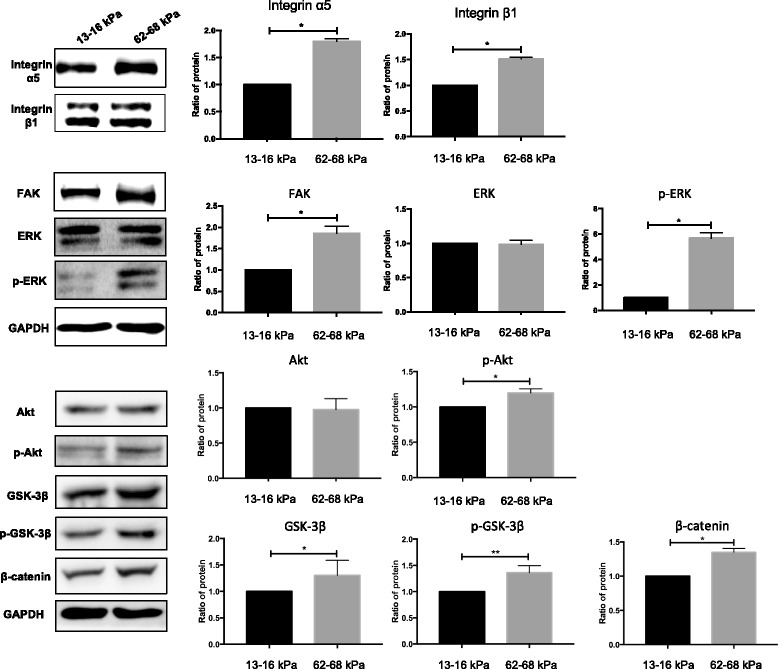
Integrin α5/β1 expression analysis of total proteins during osteogenic differentiation. Protein levels in whole cell lysates determined by western blotting. *P < 0.05. **P < 0.01. *n* = 3. ERK extracellular regulated protein kinases, FAK focal adhesion kinase, GAPDH glyceraldehyde 3-phosphate dehydrogenase, GSK3β glycogen synthase kinase 3β

Expression analysis by western blotting revealed significant increases in integrin α5 and integrin β1 levels in hMSCs cultured on 62–68 kPa ECM, suggesting that integrin β1 was likely to be internalized in these cells, which may be related to its role in intracellular signaling. When we detected the FAK–ERK–PI3K pathway, results showed that the expression of FAK, p-ERK, and p-Akt was increased whereas expression of total ERK and Akt remained unchanged. The results suggested 62–68 kPa ECM could activate phosphorylation of ERK and Akt but had no effects on expression of total ERK and Akt. Wnt/β-catenin could have crosstalk with integrin α5, because GSK-3β, p-GSK-3β, β-catenin and integrin α5 expression levels were all upregulated on 62–68 kPa ECM (Fig. 6). We then studied the links among these molecules further.

### Integrin α5-mediated osteogenic differentiation induced by matrix and expression of signaling proteins has changed after blocking integrin α5

**Fig. 7:**
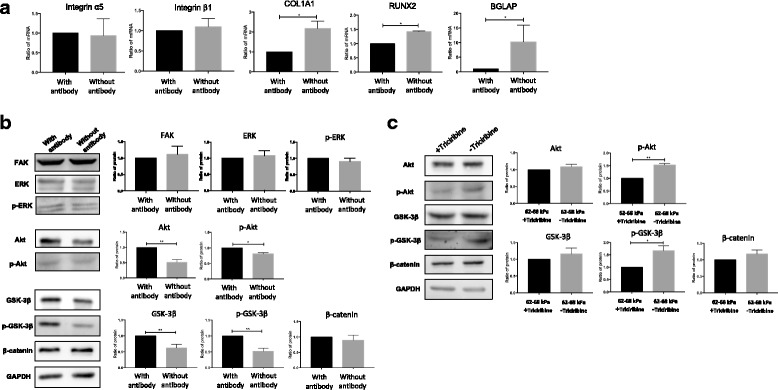
Integrin α5/β1 and osteogenic marker expression in the presence or absence of integrin α5 blocking antibody and Akt inhibitor Triciribine. **a** Integrin and osteogenic marker expression assessed by qRT-PCR. **b** Downstream signaling protein expression of integrins monitored by western blotting and quantified by densitometry. **c** Signaling protein expression detected by western blotting and quantified by densitometry with or without Triciribine. *P < 0.05. **P < 0.01. *n* = 3. COLIAI alpha-1 chains of type I collagen, RUNX2 Runt related transcription factor 2, ERK extracellular regulated protein kinases, FAK focal adhesion kinase, GAPDH glyceraldehyde 3-phosphate dehydrogenase, GSK3β glycogen synthase kinase 3β

According to previous experimental results, only cells cultured on 62–68 kPa ECM differentiated into osteoblasts and integrin α5 increased further during the process. Thus, we studied whether integrin α5 played a role in matrix-induced osteogenic differentiation. hMSCs were cultured on 62–68 kPa ECM in the presence of integrin α5 blocking antibody. Notably, treated hMSCs showed a significant decrease in *COL1A1*, *RUNX2*, and *BGLAP* expression (Fig. 7a). However, ALP and calcium deposits had no difference between the groups with or without anti-integrin α5 antibody (Additional file 1: Figure S6). Results showed that integrin α5 could mediate gene expression of osteogenic markers but has no effect on ALP and calcium deposits. Interestingly, Akt, and p-Akt were upregulated with anti-integrin α5 antibody, but no differences were observed in FAK, ERK, and p-ERK between the groups with or without anti-integrin α5 antibody. At the same time, GSK-3β and p-GSK-3β were upregulated and β-catenin levels had no difference between the groups with or without anti-integrin α5 antibody (Fig. 7b). Since Akt, p-Akt, GSK-3β, and p-GSK-3β displayed the same changes between the groups with or without anti-integrin α5 antibody, then we detected the links among them. Expression of p-Akt and p-GSK-3β was reduced effectively in the presence of the Akt inhibitor Triciribine. However, Akt, GSK-3β, and β-catenin were unchanged. These results suggested that expression of p-GSK-3β was regulated by p-Akt on 62–68 kPa ECM (Fig. 7c).

## Discussion

MSC responses to variations in ECM stiffness have been studied previously in mice [8, 34], rats [35], and humans [36, 37]. The present study examined this phenomenon in hMSCs to provide an accurate theoretical basis for clinical treatment. hMSCs are often subjected to complex interactions to induce osteogenic differentiation, including chemical and physical stimuli [38]. Because it is difficult to provide factors in vivo at the high concentrations that they are often provided *in vitro* and in a localized manner, this work focused solely on the ability of ECM stiffness to affect hMSC differentiation.

Cell morphology differs based on ECM stiffness [39, 40]. Consistently we showed that hMSCs displayed an oval, adipocyte-like appearance when cultured on 13–16 kPa ECM, but they presented a polygonal, osteoblastic morphology on 62–68 kPa ECM (Fig. 2a). However, another report found no association between ECM stiffness and MSC morphology, although similar effects were observed for osteogenic marker expression [14]. Thus, the connection among ECM stiffness and hMSC differentiation requires further study—in particular, the surface molecular mechanisms that sensed matrix stiffness.

Integrin β1 is a key molecule involved in the cellular response to substrate stiffness and related effects on differentiation potential [14, 41]. Our results demonstrated that integrin β1 expression was unaffected by ECM stiffness, but localized to the cell surface or cytoplasm when cells were cultured on soft or stiff substrates, respectively; this is opposite to the findings of Du et al. [42]. Du et al. coated the gel coated with type 1 collagen but we used fibronectin. Because different integrins have corresponding ligand proteins, that have different effects on the differentiation of cells [43], we suggest that this difference in protein might have caused the distribution of integrin β1 to be different. Then, we explored the role of integrin α subunits in the process.

Integrin α5 plays an important role in osteogenic differentiation, consistent with results of the present study. In particular, our results suggest that integrin α5 protein was localized to the cell surface, indicating that α5 did recognize the external ligands on the cell surface. Gandavarapu et al. [44] and Hogrebe and Gooch [45] demonstrated previously that increasing the binding strength of integrin α5 to ECM by adding the peptide c (RRETAWA) and RGD fragments, and increasing the site density of integrin α5, can effectively induce osteogenesis in cells cultured on the stiff ECM substrate. Increased integrin α5 could improve osteogenic differentiation of hMSCs.

Integrin α5 interacts with several signaling molecules [46], including FAK and ERK, which play important regulatory roles in matrix-induced osteogenic differentiation and gene expression [47]. Similar to the results of previous studies, our results indicated that FAK and ERK expression was markedly increased on 62–68 kPa ECM, but was not affected by blockade of integrin α5. When hMSCs were cultured in osteogenic medium on tunable polyacrylamide hydrogels, ROCK, FAK, and ERK1/2 expression was altered upon knockdown of integrin α2 by siRNA [48]. These results are different from our results possibly because we only manipulated ECM stiffness to affect hMSC differentiation. Mechanism of osteogenic differentiation would be different with changing environment.

In comparison, expression of PI3K could regulate hMSC osteogenic differentiation. They were elevated during osteogenesis induced by dexamethasone or low-intensity ultrasound, and PI3K/Akt played a critical role in this process [34, 49]. Previous reports are consistent with our results showing increased p-Akt expression during matrix-induced osteogenic differentiation. In vitro, osteoclast supernatant can also regulate osteoblast proliferation and differentiation through PI3K/Akt [50]. However, a few studies have found that PI3K/Akt signaling inhibits osteogenic differentiation [51], and these studies have focused on targeted differentiation in cells such as the precursor osteoblast cell line MC3T3-E1. In this study, hMSCs were obtained from healthy subjects to explore the effect of Akt and p-Akt signaling on the osteogenic differentiation of hMSCs, and detected significant increases in p-Akt and osteogenic differentiation on the 62–68 kPa ECM. In summary, PI3K/Akt signaling is involved in hMSC osteogenic differentiation. Osteogenic differentiation is accompanied by the expression of Akt protein downstream of the signaling pathway. In our follow-up experiments, Akt and p-Akt were increased after we blocked integrin α5. This is counter to the previous reports. We believe that when our blocking antibody blocks integrin α5, the blocked protein site may trigger a signaling molecule that activates Akt, resulting in increased Akt expression. This confirms that Akt is not regulated by integrin α5 alone and that it may be under the control of other signaling proteins.

Previous studies have shown that Wnt signaling is responsive to matrix stiffness [52]. Microarray screening results have revealed a significant promotion of the canonical Wnt/β-catenin pathway by stiffer ECM, which was confirmed by Du et al. [53]. The Wnt/β-catenin pathway can control diverse cell behaviors including cell adhesion, migration, differentiation, and proliferation, behaviors which respond significantly to ECM stiffness [26]. Inhibition of Akt has been shown to only partially block the effect of ECM stiffness on the β-catenin pathway [53], indicating that Akt contributes to but is not required for this process. Our results showed that with inhibition of Akt, β-catenin levels did not change. The integrin-activated β-catenin pathway can promote the Wnt signal by ECM stiffness and the regulation of hMSC differentiation by forming a positive feedback loop [53]. Our results showed that the promotion of the canonical Wnt/β-catenin pathway was not dependent on stiffness *per se*, but was caused by the accumulation of β-catenin.

Wnt proteins transduce their signals through disheveled proteins to inhibit GSK-3β, leading to accumulation of cytosolic β-catenin [26, 54, 55]. Regarding the role of integrins in the regulation of Wnt signaling, inhibition of integrin α5 by a functional blocking antibody significantly increased the levels of phosphorylated GSK-3β, but not those of β-catenin. As an important downstream element of integrin signaling, the FAK/Akt pathway is well documented as a regulator of GSK-3β [56, 57]. We found that 62–68 kPa ECM could increase the expression of β-catenin and phosphorylated GSK-3β. Accumulation of β-catenin was not mediated by Akt activity, as an Akt inhibitor blocked the differences in the levels of β-catenin between the stiff and the soft ECMs.

## Conclusions

Our study confirmed that hMSC culture on 62–68 kPa ECM induced osteogenic differentiation in a manner dependent of integrin α5, Akt, and GSK-3β. These results provide a theoretical basis for osteogenic hMSC differentiation of stem cells and highlight a new research direction for further studies of osteogenic differentiation and energy metabolism.

## Additional file

Additional file 1: **Figure S1.** showing identification of hMSCs. (A) Morphological appearance of third-passage hMSCs. Scale bar = 200 μm; 20 μm. (B) Logarithmic proliferation of cells. (C) Chromosome karyotype analysis of cells. (D) MSC cell surface markers evaluated through flow cytometric analysis. (E) Immunofluorescence performed using monoclonal antibodies. (F) Differentiation of hMSCs into adipocytes and osteogenic cells. Scale bar = 100 μm. *n* = 3. **Figure S2.** showing morphology of hMSCs on gels with various stiffnesses. After hMSCs were planted on the gels, the cells were analyzed with an inverted phase-contrast microscope at 4–72 h. Scale bar = 20 μm. *n* = 3. **Figure S3.** showing Phalloidin stained F-actin to examine the arrangement of the cytoskeleton, observed by confocal microscope. **Figure S4.** showing cells cultured on 13–16 kPa ECM, 62–68 kPa ECM, and TCP with cells cultured in medium and osteogenic medium at 1 week, then stained by Alizarin Red to detect calcium deposits. *n* = 3. **Figure S5.** showing hMSCs cultured on different stiffness matrices to observe expression of active integrin β1 by confocal microscope. Scale bar = 20 μm. **Figure S6.** showing cells cultured on 13–16 kPa ECM, 62–68 kPa ECM, and TCP with or without anti-integrin α5 antibody for 1 week, then observing ALP expression and calcium deposits. Scale bar = 200 μm (PDF 19990 kb)

## Abbreviations

ALP: Alkaline phosphatase
bFGF: Basic fibroblast growth factor
COLIAI: Alpha-1 chains of type I collagen
DMSO: Dimethyl sulfoxide
ECM: Extracellular matrix
ERK: Extracellular regulated protein kinases
FAK: Focal adhesion kinase
FBS: Fetal bovine serum
GSK3β: Glycogen synthase kinase 3β
HEPES: 2-(4-(2-Hydroxyethyl)piperazin-1-yl)ethanesulfonic acid
hMSC: Human mesenchymal stem cell
IGF-1: Insulin-like growth factors
PI3K: Phosphoinositide 3-kinase
RGD: Arg-Gly-Asp
RUNX2: Runt related transcription factor 2
S6 K1: Kinase for pSer240/244 S6
SEM: Scanning electron microscope
TCP: Tissue culture plates
TORC1: The target of rapamycin complex 1
UV: Ultraviolet ray

## References

[1] Bianco P (2008). Mesenchymal stem cells: revisiting history, concepts, and assays. Cell Stem Cell.

[2] Lv HW (2015). Union is strength: matrix elasticity and microenvironmental factors codetermine stem cell differentiation fate. Cell and tissue research.

[3] Satue M, Ramis JM, Monjo M (2016). UV-activated 7-dehydrocholesterol-coated titanium implants promote differentiation of human umbilical cord mesenchymal stem cells into osteoblasts. J Biomater Appl.

[4] Ghali O (2015). Dexamethasone in osteogenic medium strongly induces adipocyte differentiation of mouse bone marrow stromal cells and increases osteoblast differentiation. BMC Cell Biol.

[5] Baumgart E (2000). Stiffness—an unknown world of mechanical science?. Injury.

[6] Lo WJ (2000). Physical, chemical, and biological characterization of pulsed laser deposited and plasma sputtered hydroxyapatite thin films on titanium alloy. J Biomed Mater Res.

[7] Tskhovrebova L, Trinick J (2001). Flexibility and extensibility in the titin molecule: analysis of electron microscope data. J Mol Biol.

[8] Lv HW, et al. Biomaterial stiffness determines stem cell fate. Life sciences. 2017;178:42-8. 10.1016/j.lfs.2017.04.014 28433510

[9] Popov C (2011). Integrins α2β1 and α11β1 regulate the survival of mesenchymal stem cells on collagen I. Cell Death Dis.

[10] Veevers-Lowe J (2011). Mesenchymal stem cell migration is regulated by fibronectin through α5β1-integrin-mediated activation of PDGFR-β and potentiation of growth factor signals. J Cell Sci.

[11] Zou C (2011). Mesenchymal stem cells require integrin beta1 for directed migration induced by osteopontin in vitro. In Vitro Cell Dev Biol Anim.

[12] Hamidouche Z (2009). Priming integrin α5 promotes human mesenchymal stromal cell osteoblast differentiation and osteogenesis. Proc Natl Acad Sci U S A.

[13] Gandavarapu NR, Alge DL, Anseth KS (2014). Osteogenic differentiation of human mesenchymal stem cells on α5 integrin binding peptide hydrogels is dependent on substrate elasticity. Biomater Sci.

[14] Olivares-Navarrete R (2017). Substrate stiffness controls osteoblastic and chondrocytic differentiation of mesenchymal stem cells without exogenous stimuli. PLoS One.

[15] Srouji S (2012). Lentiviral-mediated integrin alpha5 expression in human adult mesenchymal stromal cells promotes bone repair in mouse cranial and long-bone defects. Hum Gene Ther.

[16] Fromigue O (2012). Peptide-based activation of alpha5 integrin for promoting osteogenesis. J Cell Biochem.

[17] Di Benedetto A (2015). Osteogenic differentiation of mesenchymal stem cells from dental bud: role of integrins and cadherins. Stem Cell Res.

[18] Fraioli R (2015). Mimicking bone extracellular matrix: integrin-binding peptidomimetics enhance osteoblast-like cells adhesion, proliferation, and differentiation on titanium. Colloids Surf B Biointerfaces.

[19] Saidak Z (2015). Wnt/β-catenin signaling mediates osteoblast differentiation triggered by peptide-induced α5β1 integrin priming in mesenchymal skeletal cells. J Biol Chem.

[20] Gu YX (2013). The roles of PI3K/Akt signaling pathway in regulating MC3T3-E1 preosteoblast proliferation and differentiation on SLA and SLActive titanium surfaces. J Biomed Mater Res A.

[21] Liu X (2007). Lifelong accumulation of bone in mice lacking Pten in osteoblasts. Proc Natl Acad Sci U S A.

[22] Hendesi H (2015). Integrin mediated adhesion of osteoblasts to connective tissue growth factor (CTGF/CCN2) induces cytoskeleton reorganization and cell differentiation. PLoS One.

[23] Hsu YJ (2015). Thiazide-sensitive Na+-Cl– cotransporter (NCC) gene inactivation results in increased duodenal Ca2+ absorption, enhanced osteoblast differentiation and elevated bone mineral density. J Bone Miner Res.

[24] Chim SM (2015). EGFL7 is expressed in bone microenvironment and promotes angiogenesis via ERK, STAT3, and integrin signaling cascades. J Cell Physiol.

[25] Lambertini E (2015). Osteogenic differentiation of human MSCs: specific occupancy of the mitochondrial DNA by NFATc1 transcription factor. Int J Biochem Cell Biol.

[26] Clevers H (2006). Wnt/β-catenin signaling in development and disease. Cell.

[27] Wang Y (2014). Wnt and the Wnt signaling pathway in bone development and disease. Front Biosci (Landmark Ed).

[28] Ma DH (2016). Preservation of human limbal epithelial progenitor cells on carbodiimide cross-linked amniotic membrane via integrin-linked kinase-mediated Wnt activation. Acta Biomater.

[29] Happe CL, Engler AJ (2016). Mechanical forces reshape differentiation cues that guide cardiomyogenesis. Circ Res.

[30] Olivares-Navarrete R (2011). Mediation of osteogenic differentiation of human mesenchymal stem cells on titanium surfaces by a Wnt-integrin feedback loop. Biomaterials.

[31] Ozeki N (2016). Autophagy-related gene 5 and Wnt5 signaling pathway requires differentiation of embryonic stem cells into odontoblast-like cells. Exp Cell Res.

[32] Li Q (2009). Extracellular matrix with the rigidity of adipose tissue helps 3T3-L1 adipocytes maintain insulin responsiveness. J Med Invest.

[33] Asghari Sana F (2017). Spreading, proliferation and differentiation of human dental pulp stem cells on chitosan scaffolds immobilized with RGD or fibronectin. Cytotechnology.

[34] Watabe H (2011). Mechanotransduction activates alpha(5)beta(1) integrin and PI3K/Akt signaling pathways in mandibular osteoblasts. Exp Cell Res.

[35] Jiang P, Mao Z, Gao C (2015). Combinational effect of matrix elasticity and alendronate density on differentiation of rat mesenchymal stem cells. Acta Biomater.

[36] Xu JJ (2017). Effect of matrix stiffness on the proliferation and differentiation of umbilical cord mesenchymal stem cells. Differentiation..

[37] Lee J (2016). Matrix directed adipogenesis and neurogenesis of mesenchymal stem cells derived from adipose tissue and bone marrow. Acta Biomater.

[38] Lv HW (2015). Mechanism of regulation of stem cell differentiation by matrix stiffness. Stem cell research & therapy..

[39] Geiger B, Spatz JP, Bershadsky AD (2009). Environmental sensing through focal adhesions. Nat Rev Mol Cell Biol.

[40] Sun MY (2018). Effects of Matrix Stiffness on the Morphology, Adhesion, Proliferation and Osteogenic Differentiation of Mesenchymal Stem Cells. Int J Med Sci.

[41] Frith JE (2012). Tailored integrin-extracellular matrix interactions to direct human mesenchymal stem cell differentiation. Stem Cells Dev.

[42] Du J (2011). Integrin activation and internalization on soft ECM as a mechanism of induction of stem cell differentiation by ECM elasticity. Proc Natl Acad Sci U S A.

[43] Mathews S (2012). Extracellular matrix protein mediated regulation of the osteoblast differentiation of bone marrow derived human mesenchymal stem cells. Differentiation.

[44] Gandavarapu NR, Alge DL, Anseth KS (2014). Osteogenic differentiation of human mesenchymal stem cells on alpha 5 integrin binding peptide hydrogels is dependent on substrate elasticity (vol 2, pg 352, 2014). Biomater Sci.

[45] Hogrebe NJ, Gooch KJ (2016). Direct influence of culture dimensionality on human mesenchymal stem cell differentiation at various matrix stiffnesses using a fibrous self-assembling peptide hydrogel. J Biomed Mater Res A.

[46] Hynes RO (2002). Integrins: bidirectional, allosteric signaling machines. Cell.

[47] Hamidouche Z (2009). Priming integrin α5 promotes human mesenchymal stromal cell osteoblast differentiation and osteogenesis. Bone.

[48] Shih YR (2011). Matrix stiffness regulation of integrin-mediated mechanotransduction during osteogenic differentiation of human mesenchymal stem cells. J Bone Miner Res.

[49] Hamidouche Z (2010). Crosstalks between integrin alpha 5 and IGF2/IGFBP2 signalling trigger human bone marrow-derived mesenchymal stromal osteogenic differentiation. BMC Cell Biol.

[50] Chen LL (2013). PI3K/AKT pathway involvement in the osteogenic effects of osteoclast culture supernatants on preosteoblast cells. Tissue Eng Part A.

[51] Zhang Y, Yang JH (2013). Activation of the PI3K/Akt pathway by oxidative stress mediates high glucose-induced increase of adipogenic differentiation in primary rat osteoblasts. J Cell Biochem.

[52] Barbolina MV (2013). Matrix rigidity activates Wnt signaling through down-regulation of Dickkopf-1 protein. J Biol Chem.

[53] Du J (2016). Extracellular matrix stiffness dictates Wnt expression through integrin pathway. Sci Rep.

[54] Ikeda S (1998). Axin, a negative regulator of the Wnt signaling pathway, forms a complex with GSK-3β and β-catenin and promotes GSK-3β-dependent phosphorylation of β-catenin. EMBO J.

[55] Liu C (2002). Control of β-catenin phosphorylation/degradation by a dual-kinase mechanism. Cell.

[56] Romorini L (2016). AKT/GSK3β signaling pathway is critically involved in human pluripotent stem cell survival. Sci Rep.

[57] Su YJ (2015). Polarized cell migration induces cancer type-specific CD133/integrin/Src/Akt/GSK3β/β-catenin signaling required for maintenance of cancer stem cell properties. Oncotarget.

