# Supplementary material for: Extracellular matrix stiffness controls osteogenic differentiation of mesenchymal stem cells mediated by integrin α5
Source: Stem Cell Res Ther. 2018 Mar 1;9:52. doi: 10.1186/s13287-018-0798-0 (PMC5831741; doi:10.1186/s13287-018-0798-0)
Supplement: Supplementary file 1 — Figure S1. showing identification of hMSCs. (A) Morphological appearance of third-passage hMSCs. Scale bar = 200 μm; 20 μm. (B) Logarithmic proliferation of cells. (C) Chromosome karyotype analysis of cells. (D) MSC cell surface markers evaluated through flow cytometric analysis. (E) Immunofluorescence performed using monoclonal antibodies. (F) Differentiation of hMSCs into adipocytes and osteogenic cells. Scale bar = 100 μm. n = 3. Figure S2. showing morphology of hMSCs on gels with various stiffnesses. After hMSCs were planted on the gels, the cells were analyzed with an inverted phase-contrast microscope at 4–72 h. Scale bar = 20 μm. n = 3. Figure S3. showing Phalloidin stained F-actin to examine the arrangement of the cytoskeleton, observed by confocal microscope. Figure S4. showing cells cultured on 13–16 kPa ECM, 62–68 kPa ECM, and TCP with cells cultured in medium and osteogenic medium at 1 week, then stained by Alizarin Red to detect calcium deposits. n = 3. Figure S5. showing hMSCs cultured on different stiffness matrices to observe expression of active integrin β1 by confocal microscope. Scale bar = 20 μm. Figure S6. showing cells cultured on 13–16 kPa ECM, 62–68 kPa ECM, and TCP with or without anti-integrin α5 antibody for 1 week, then observing ALP expression and calcium deposits. Scale bar = 200 μm (PDF 19990 kb) [file 13287_2018_798_MOESM1_ESM.pdf]

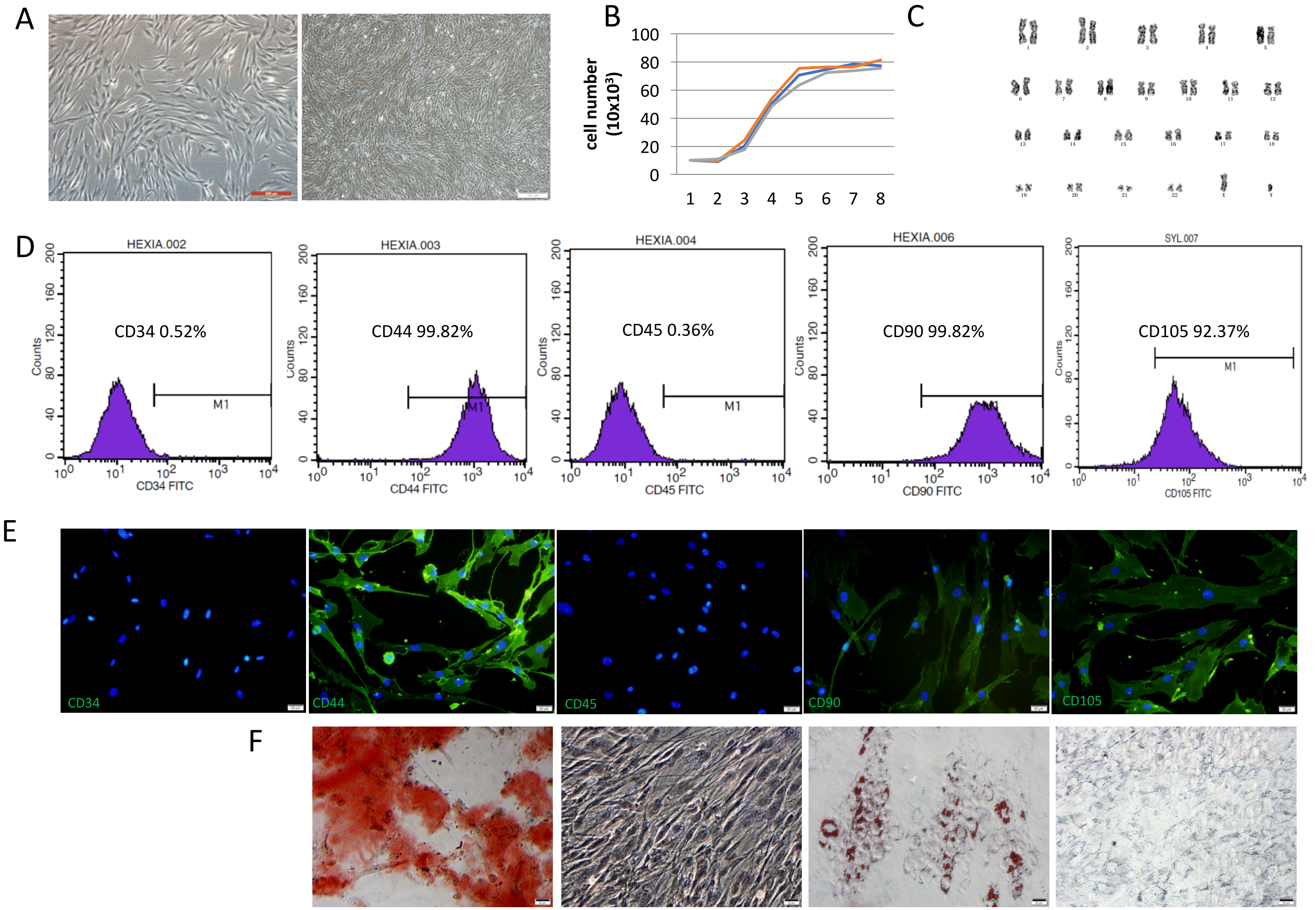

A
B
cell number (10x10³)
100
80
60
40
20
0
1 2 3 4 5 6 7 8
C
D
HEXIA.002
CD34 0.52%
Counts
CD34 FITC
HEXIA.003
CD44 99.82%
CD44 FITC
HEXIA.004
CD45 0.36%
CD45 FITC
HEXIA.006
CD90 99.82%
CD90 FITC
SYL.007
CD105 92.37%
CD105 FITC
M1
E
CD34
CD44
CD45
CD90
CD105
F

S1

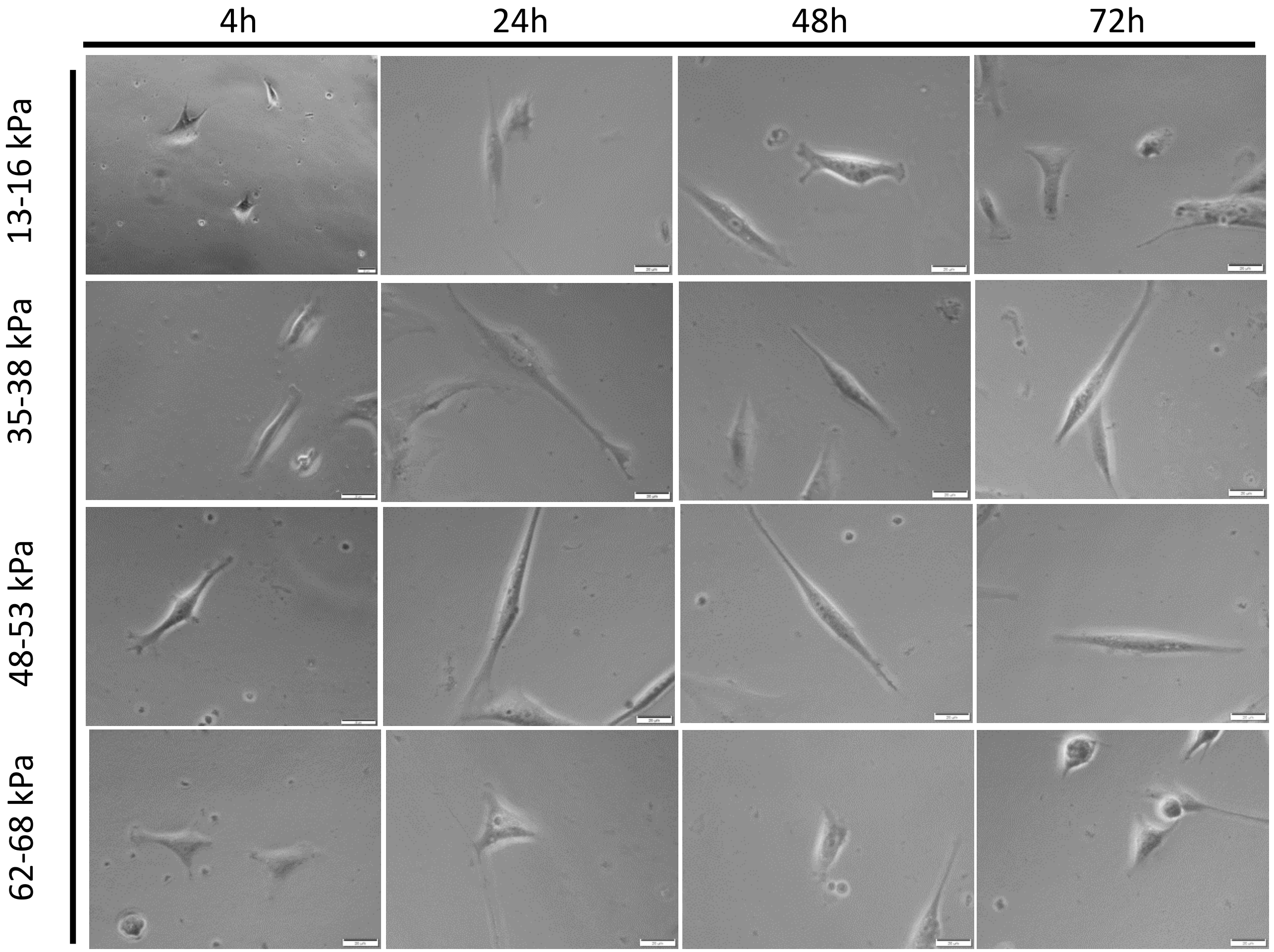

S2

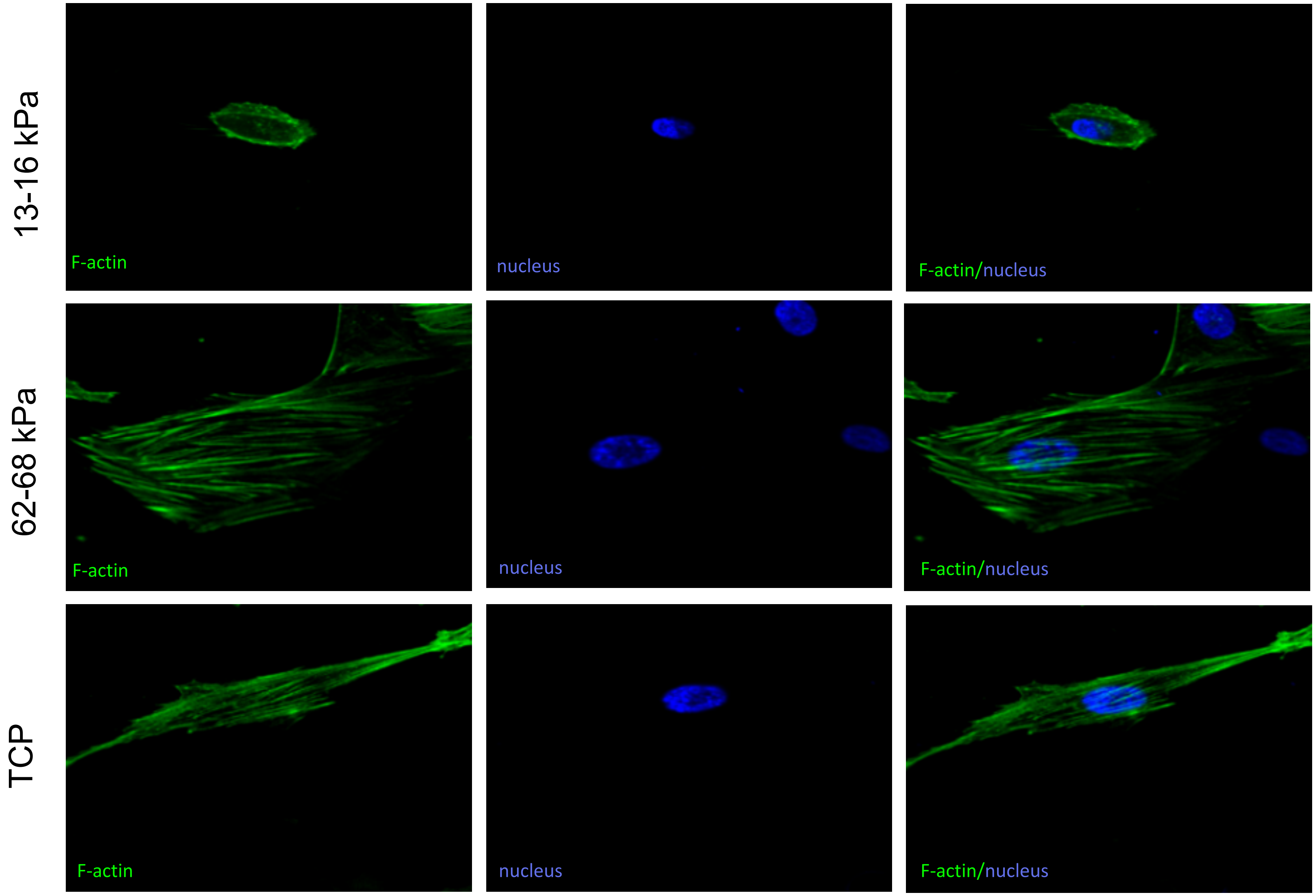
13-16 kPa
62-68 kPa
TCP
F-actin
nucleus
F-actin/nucleus
F-actin
nucleus
F-actin/nucleus
F-actin
nucleus
F-actin/nucleus

S3

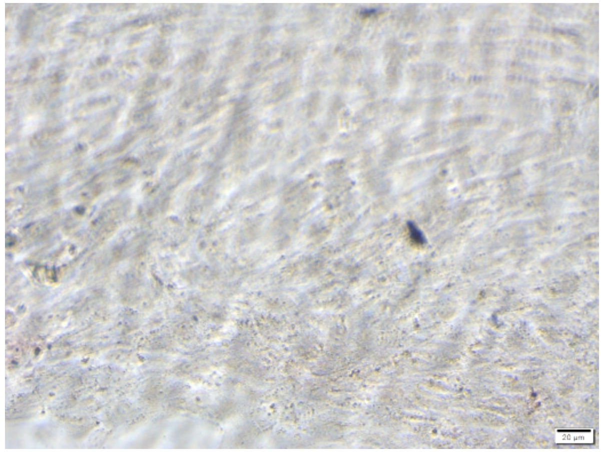
13-16 kPa

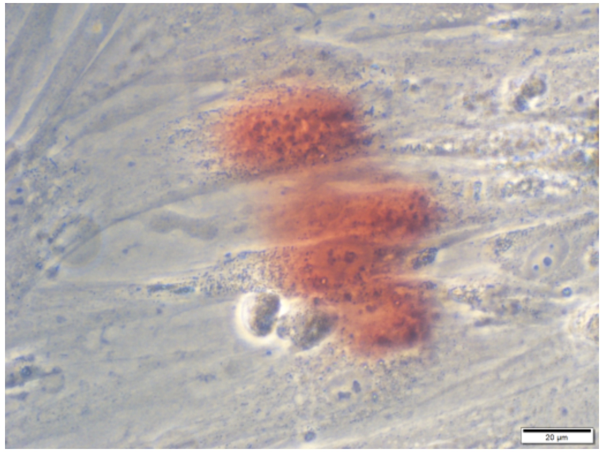
62-68 kPa

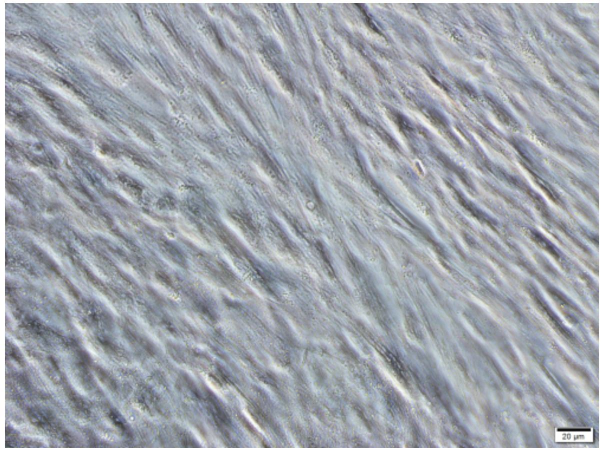
TCP

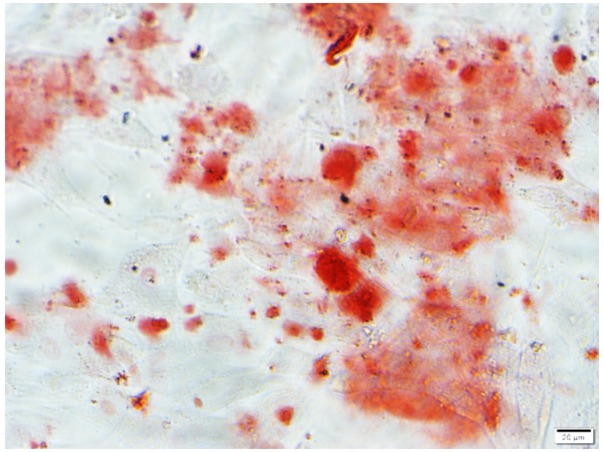
Osteogenic medium +13-16 kPa

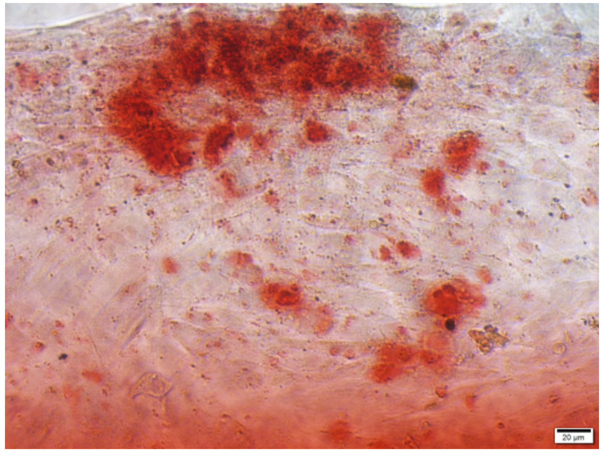
Osteogenic medium + 62-68 kPa

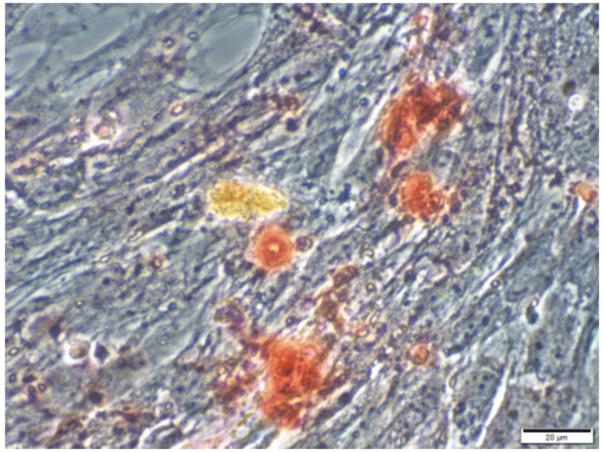
Osteogenic medium+ TCP

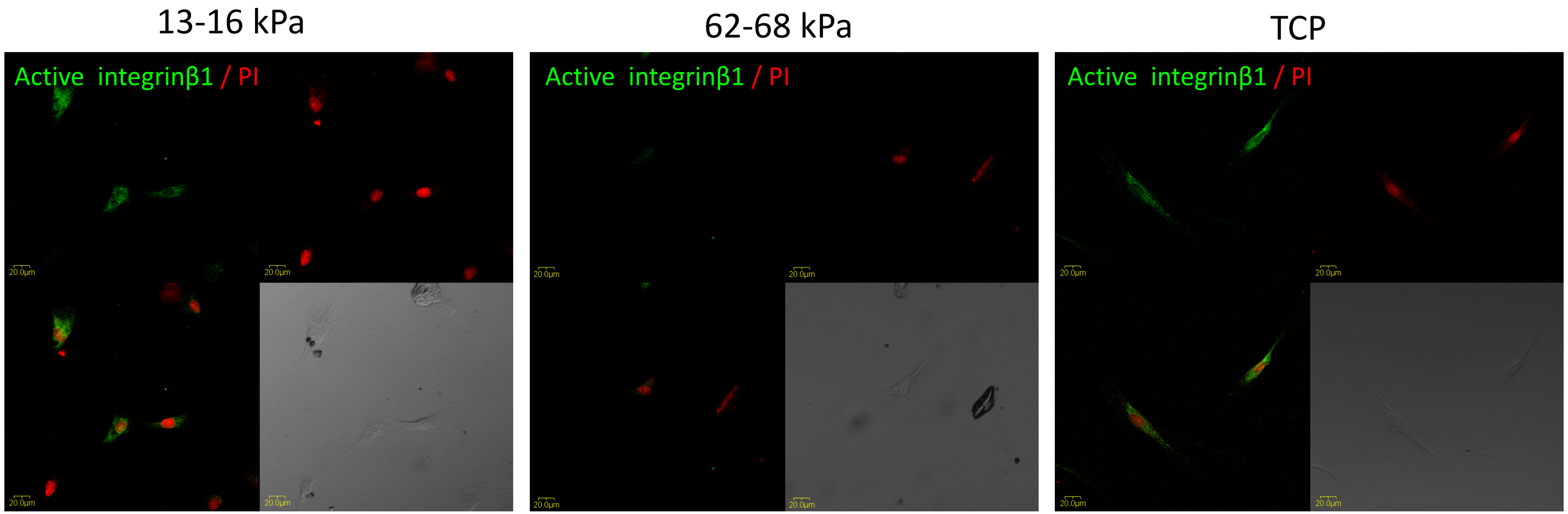
13-16 kPa
Active integrinβ1 / PI
62-68 kPa
Active integrinβ1 / PI
TCP
Active integrinβ1 / PI

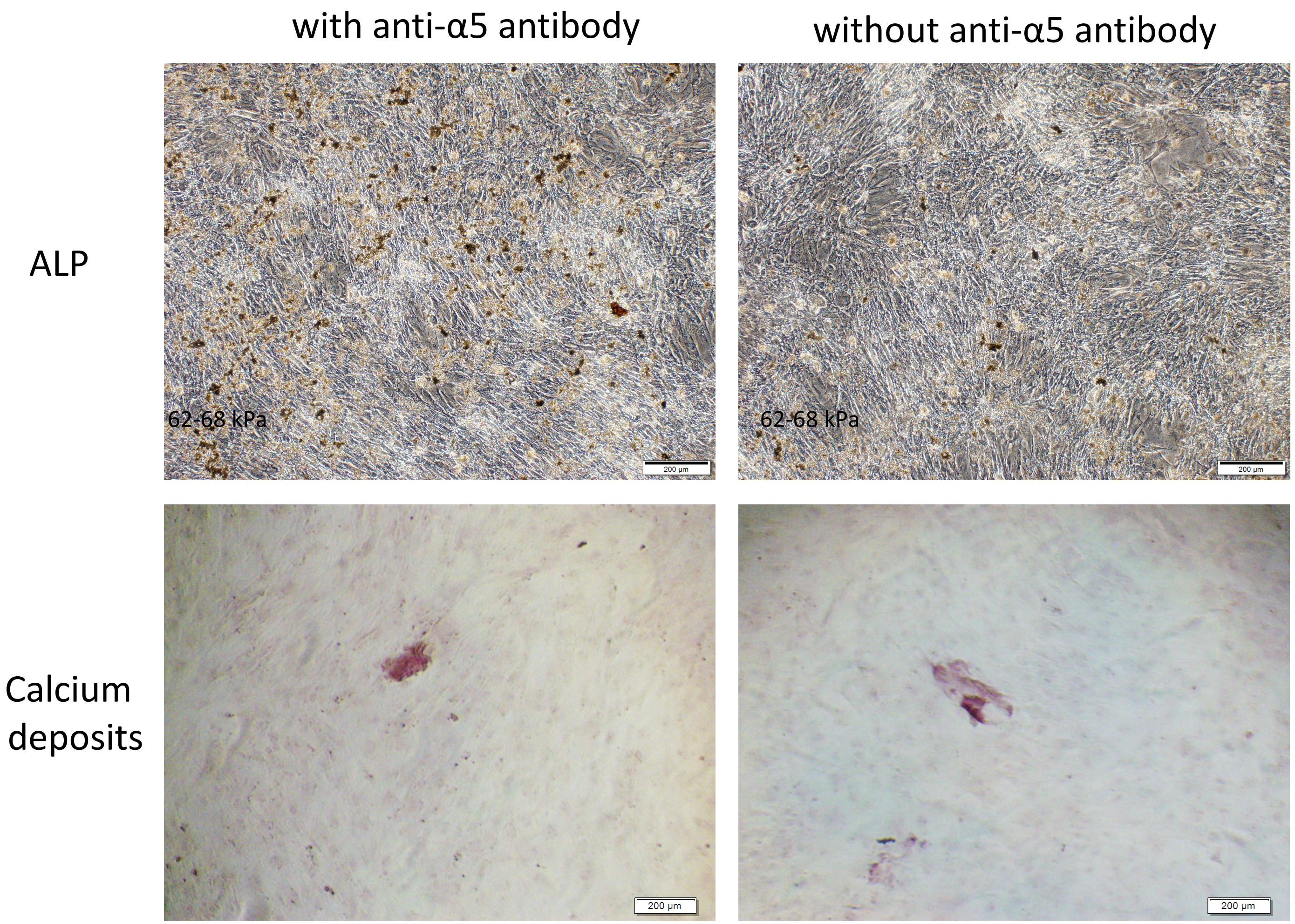
with anti-α5 antibody
without anti-α5 antibody
ALP
Calcium deposits
62-68 kPa
62-68 kPa
200 µm
200 µm
200 µm
200 µm
